# CAR-NK Cells Effectively Target SARS-CoV-2-Spike-Expressing Cell Lines *In Vitro*

**DOI:** 10.3389/fimmu.2021.652223

**Published:** 2021-07-23

**Authors:** Minh Tuyet Ma, Saiaditya Badeti, Chih-Hsiung Chen, James Kim, Alok Choudhary, Bill Honnen, Charles Reichman, David Calianese, Abraham Pinter, Qingkui Jiang, Lanbo Shi, Renping Zhou, Huanbin Xu, Qingsheng Li, William Gause, Dongfang Liu

**Affiliations:** ^1^ Department of Pathology, Immunology and Laboratory Medicine, Newark, NJ, United States; ^2^ School of Graduate Studies, Rutgers Biomedical and Health Sciences, Newark, NJ, United States; ^3^ Department of Microbiology, Biochemistry & Molecular Genetics, Public Health Research Institute Center, New Jersey Medical School, Rutgers University, Newark, NJ, United States; ^4^ Public Health Research Institute, New Jersey Medical School, Rutgers Biomedical and Health Sciences, Rutgers, The State University of New Jersey, Newark, NJ, United States; ^5^ Department of Chemical Biology, Ernest Mario School of Pharmacy, Rutgers University, Piscataway, NJ, United States; ^6^ Division of Comparative Pathology, Tulane National Primate Research Center, Covington, LA, United States; ^7^ Nebraska Center for Virology and School of Biological Sciences, University of Nebraska, Lincoln, NE, United States; ^8^ Center for Immunity and Inflammation, New Jersey Medical School, Rutgers-The State University of New Jersey, Newark, NJ, United States

**Keywords:** CAR (chimeric antigen receptor), NK cells, COVID-19, SARS-CoV-2, N501Y variant, E484K variant, off-the-shelf

## Abstract

Severe Acute Respiratory Syndrome Coronavirus 2 (SARS-CoV-2) is highly contagious and presents a significant public health issue. Current therapies used to treat coronavirus disease 2019 (COVID-19) include monoclonal antibody cocktail, convalescent plasma, antivirals, immunomodulators, and anticoagulants. The vaccines from Pfizer and Moderna have recently been authorized for emergency use, which are invaluable for the prevention of SARS-CoV-2 infection. However, their long-term side effects are not yet documented, and populations with immunocompromised conditions (e.g., organ-transplantation and immunodeficient patients) may not be able to mount an effective immune response. In addition, there are concerns that wide-scale immunity to SARS-CoV-2 may introduce immune pressure that could select for escape mutants to the existing vaccines and monoclonal antibody therapies. Emerging evidence has shown that chimeric antigen receptor (CAR)- natural killer (NK) immunotherapy has potent antitumor response in hematologic cancers with minimal adverse effects in recent studies, however, the potentials of CAR-NK cells in treating COVID-19 has not yet been fully exploited. Here, we improve upon a novel approach for the generation of CAR-NK cells for targeting SARS-CoV-2 and its various mutants. CAR-NK cells were generated using the scFv domain of S309 (henceforward, S309-CAR-NK), a SARS-CoV and SARS-CoV-2 neutralizing antibody (NAbs) that targets the highly conserved region of SARS-CoV-2 spike (S) glycoprotein and is therefore more likely to recognize different variants of SARS-CoV-2 isolates. S309-CAR-NK cells can specifically bind to pseudotyped SARS-CoV-2 virus and its D614G, N501Y, and E484K mutants. Furthermore, S309-CAR-NK cells can specifically kill target cells expressing SARS-CoV-2 S protein *in vitro* and show superior killing activity and cytokine production, compared to that of the recently reported CR3022-CAR-NK cells. Thus, these results pave the way for generating ‘off-the-shelf’ S309-CAR-NK cells for treatment in high-risk individuals as well as provide an alternative strategy for patients unresponsive to current vaccines.

## Introduction

SARS-CoV-2 is highly transmissible and has thus far infected millions of people worldwide with the U.S. making up approximately one-fifth of total reported cases (1). Since February 2020, the frequency of the SARS-CoV-2 D614G, N501Y, and E484K variants have increased significantly and has become the dominant variant over the course of the pandemic (2). The D614G variant is shown to associate with higher viral loads in patients, though there is currently insufficient scientific evidence showing the effect of D614G mutation in increased infectivity and transmissibility (3, 4). The disease caused by SARS-CoV-2 (i.e., COVID-19) presents severe symptoms including pneumonia, acute respiratory distress syndrome (5), neurological symptoms, organ failure, and death. More importantly, severe COVID-19 patients may experience dysregulation of an appropriate immune response, characterized by lymphopenia (6), high neutrophil levels in peripheral blood (7), and increased pro-inflammatory cytokines and chemokines (8). Repurposed therapeutics such as monoclonal antibody cocktails, convalescent plasma, and dexamethasone have shown promising results in treating COVID-19 (9). The Pfizer/BioNTech (NCT04368728) and Moderna (NCT04283461) vaccines were recently approved by the FDA for emergency use (10, 11); however, their long-term effects have not yet been documented (12–14). Furthermore, there is accumulating evidence that viral variants that are relatively resistant to current vaccines and antibody treatments are becoming dominant. There could also be subsets of patients who may not be responsive to the vaccines (e.g., people with immune-compromising or immunodeficiency conditions), underscoring the need for an alternative protection or treatment strategy in preparation for future pandemics. Future SARS-CoV-2-related pandemics might be inevitable. Preparing for the next global pandemic requires vaccines, as well as various treatment strategies. Engineering memory-like, long-lived, highly potent, ‘off-the-shelf’, SARS-CoV-2 virus specific CAR-NK cells represents one treatment strategy and is essential for preparing for the next global pandemic.

Multiple sources of NK cells are found in cord blood (CB), peripheral blood (PB), bone marrow (BM), human embryonic stem cells (ESCs), and induced pluripotent stem cells (iPSC) (15, 16). NK cells isolated from these sources can be further modified to express a chimeric antigen receptor (CAR) for treating a variety of cancers and infectious diseases (17). Recent preclinical studies of CAR-NK in cancer immunotherapy show several advantages of CAR-NK cells over CAR-T cells in clinical safety. For instance, CAR-NK cells do not present additional risk for the development of severe graft-versus-host-disease (GVHD) (18). More importantly, CAR-NK cells are associated with reduced host cytotoxicity compared to CAR-T cells. Specifically, NK cells are less likely to induce cytokine release syndrome (CRS) that could potentially exacerbate COVID-19 symptoms in severe patients (19). Additionally, CAR-NK cells have potential to be developed as a ‘off-the-shelf’ CAR product in the near future (17, 18, 20). Given these aforementioned reasons, NK and CAR-NK cell-based immunotherapeutics have been rapidly developed for COVID-19 treatment. Specifically, adoptive transfer of monocytes or NK cells (NCT04797975, NCT04280224, and NCT04365101) and the universal ‘off-the-shelf’ NKG2D-ACE2 CAR-NK cells (NCT04324996) expanded from CB are currently being studied in clinical trials for COVID-19.

Previous studies show that the genome sequence of SARS-CoV-2 is 79.6% identical to that of SARS-CoV (21). Similar to SARS-CoV, the Spike protein expressed on the surface of SARS-CoV-2 binds to the angiotensin-converting enzyme-2 (ACE2) receptor and facilitates virus entry (21, 22). Several NAbs were isolated from memory B cells of convalescent SARS patients which possess cross-reactivity for SARS-CoV-2. One such antibody, named S309, potently neutralizes both pseudotyped SARS-CoV-2 viral particles and wild-type SARS-CoV-2 by binding to both the ‘closed’ and ‘open’ ectodomain trimer conformations of the SARS-CoV-2 Spike glycoprotein (23). Therefore, engineering NK cells to express CARs that both recognize SARS-CoV-2 and activate NK cells is feasible.

In this study, we developed a novel approach for the generation of CAR-NK cells for targeting SARS-CoV-2 using the scFv domain of S309. Here, we show that both the S309-CAR-NK-92MI cell line and primary S309-CAR-NK cells expanded from PB (hereinafter S309-CAR-NK^primary^) cells effectively bind to SARS-CoV-2 pseudovirus and the D614G variant pseudovirus. Moreover, compared to the previously generated Spike-protein-targeting CR3022-CAR-NK cells (24), S309-CAR-NK cells show superior killing activities against target cells [e.g., A549, an epithelial carcinoma derived from a 58 year old Caucasian male with a non-small cell lung carcinoma (25)] expressing SARS-CoV-2 S protein and mutant S protein (hereinafter, A549-Spike and A549-Spike D614G, respectively) *in vitro*. S309-CAR-NK^primary^ cells show increased productions of TNF-α and IFN-γ when cocultured with A549-Spike and A549-Spike D614G target cells in comparison to CR3022-CAR-NK^primary^. The “4-hour gold standard” chromium release assay further confirmed the superior killing activities of both S309-CAR-NK-92MI cell line and S309-CAR-NK^primary^. These data show that ‘off-the-shelf’ S309-CAR-NK cells may have the potential to treat immunocompromised patients or those with comorbidities such as diabetes, cancer, malnutrition, and certain genetic disorders who have been infected with SARS-CoV-2. Thus, engineering highly potent, ‘off-the-shelf’, SARS-CoV-2 virus specific-CAR-NK cells represents a valid novel treatment strategy and which is essential for preparing for the next global pandemic.

## Materials and Methods

### Antibodies and Cell Lines

The information about the antibodies used in this study is provided by **Table S1**. 293T, HepG2, and NK-92MI cell lines were purchased from the American Type Culture Collection (ATCC). A549 cell line is a gift from Dr. Wei-Xing Zong (Rutgers Ernest Mario School of Pharmacy). 293T, A549, and HepG2 cell lines were cultured in DMEM (Corning) supplemented with 10% fetal bovine serum (FBS), and 100 U/mL Penicillin-Streptomycin (Corning). 293T-hACE2 cell line is a gift from Dr. Abraham Pinter (Rutgers-New Jersey Medical School, PHRI). To maintain the stable expression of hACE2, 293T-hACE2 cells were cultured in DMEM supplemented with 10% FBS, 100 U/mL Penicillin-Streptomycin, and 1μg/mL of puromycin. NK-92MI cell line was cultured in NK-92MI media, which contains α−MEM media (Gibco), 1.5 g/L sodium bicarbonate, 0.2 mM inositol, 0.1 mM 2-mercaptoethanol, 0.02 mM folic acid, 12.5% horse serum (Gibco), and 12.5% FBS.

### Generation of Transient 293T-hACE2-RBD Cell Line

To establish the transient 293T-hACE2-RBD cell line, 293T-hACE2 cells were transfected with 0.5 μg of SARS-CoV-2-RBD plasmid (a gift from Dr. Abraham Pinter) and Genejuice (Biosciences) in 1 mL Opti-MEM transfection medium (ThermoFisher) in each well in a 24-well plate (Eppendorf) for 48 hours at 37°C under 5% CO_2_. Transfected cells were harvested after 48-72 hours and stained with 1:100 primary anti-RBD or anti-Spike subunit 1 (SinoBiological) followed by a goat anti-rabbit fluorophore-conjugated secondary antibody to determine the expression of RBD by flow cytometry.

### Generation of Stable A549-Spike and A549-Spike Bearing D614G, E484K, or N501Y Mutation Cell Lines

pcDNA3.1-SARS-CoV-2 Spike (Addgene plasmid #145302) was used to clone SARS-CoV-2 S gene into the SFG backbone using the In-Fusion Cloning kit (Takara Bio). pSFG-SARS-CoV-2 S D614G, E484K, or N501Y was mutagenized from pSFG-SARS-CoV-2 S plasmid using the Q5 Site-Directed Mutagenesis Kit (New England BioLabs). 293T cells were transfected with 3.75 μg pSFG-SARS-CoV-2 S or pSFG-SARS-CoV-2 S D614G or E484K or N501Y, 2.5 μg RDF plasmid, 3.75 μg PegPam3 and Genejuice in 1 mL Opti-MEM medium for 48 hours at 37°C under 5% CO_2_. The spike retrovirus supernatant was filtered (0.45 μm) and transduced into A549 cells using RetroNectin-coated 24-well plates (RetroNectin was purified in-house) for an additional 48-72 hours at 37°C under 5% CO_2_. After 2-3 days, transduced cells were continued culture in fresh DMEM supplemented with 10% FBS, 100 U/mL Penicillin-Streptomycin. The spike protein expression was determined by flow cytometry by staining the transduced cells with anti-RBD antibody or anti-Spike subunit 1 (SinoBiological) followed by a goat anti-rabbit fluorophore-conjugated secondary antibody. A549-Spike cells were cultured for a few days prior to sorting using anti-RBD or anti-Spike subunit 1. Sorted cells were cultured in DMEM supplemented with 10% FBS, and 100 U/mL Penicillin-Streptomycin.

### Production of Pseudotyped SARS-CoV-2 S Viral Particles

The Q5 Site-Directed Mutagenesis Kit was used to mutagenize pcDNA3.1-SARS-CoV-2 Spike (Addgene plasmid #145302). Briefly, 293T cells were transfected using a lentivirus system with a combination of plasmids including 1.83 μg pLP1, 2.62 μg pLP2, 4.17 μg pCMV-luciferase-ecoGFP (a gift from Dr. Pei-Hui Wang in Advanced Medical Research Institute, Cheeloo College of Medicine, Shandong University, Jinan, 250012, China), 6.4 μg pcDNA 3.1-SARS-CoV-2 Spike (Addgene plasmid #145032), and Genejuice in 1 mL Opti-MEM media for 72 hours at 37°C under 5% CO_2_. The pseudovirus was then filtered (0.45 μm). To confirm the presence of pseudotyped SARS-CoV-2 viral particles, the filtered pseudovirus supernatant was used to transfect 293T-hACE2 for 48 hours at 37°C under 5% CO_2_. The GFP expression of the transfected 293T-hACE2 cells was observed using an EVOS FL microscope (Life Technologies). The presence of the SARS-CoV-2 pseudovirus was further confirmed by flow cytometry, transfected 293T-hACE2 cells were stained with 1:100 primary anti-RBD or anti-Spike subunit 1 followed by goat anti-rabbit fluorophore-conjugated secondary antibody.

### S309-CAR Construction and Retrovirus Production

A codon-optimized DNA fragment was synthesized by GENEWIZ encoding the S309-specific scFv and sub-cloned into the SFG retroviral backbone as described (26). S309-CAR retrovirus was generated as described and subsequently transduced to NK92-MI cells. 48 – 72 hours post-transduction, cells were transferred to 75 cm^2^ flask (Corning) in complete NK-92MI medium. To determine the expression of CAR or to sort S309-CAR-NK-92MI cell line, cells were stained with anti-CD56 and anti-human IgG(H+L) F(ab’)_2_ fragment.

### Primary NK Cell Expansion From Peripheral Blood

The Rutgers University Institutional Review Board (IRB) approved the human blood related work in this manuscript. Primary NK cell expansion was performed as previously described (26).

### Generation of CAR-NK^primary^ Cells

CAR-NK^primary^ cells were expanded and generated as described previously (26).

### S309-CAR and RBD Binding Assay

To evaluate the binding activity of CR3022-CAR to RBD domain of SARS-CoV-2 S, S309-CAR or NK-92MI (5 × 10^5^) cells were incubated with 5 µg of His-gp70-RBD recombinant protein (a gift from Dr. Abraham Pinter from Rutgers-PHRI) in DPBS buffer (0.5 mM MgCl_2_ and 0.9 mM CaCl_2_ in PBS) for 30 minutes on ice. Cells were washed twice with PBS, stained with anti-His in FACS buffer (0.2% FBS in PBS) for 30 minutes on ice and then washed twice with PBS. Cells were then stained with goat anti-mouse (IgG1) secondary antibody in FACS buffer for 30 minutes on ice, washed twice with PBS, and analyzed by flow cytometry.

### S309-CAR and Pseudotyped SARS-CoV-2 S Viral Particles Binding Assay

S309-CAR, NK-92MI, and 293T-hACE2 (5 × 10^5^) cells were first equilibrated with binding media (complete RPMI-1640 containing 0.2% BSA and 10 mM HEPES pH 7.4). Due to the non-specific binding to the S309-CAR of our secondary antibody, cells were first blocked with anti-human IgG(H+L) F(ab’)_2_ fragment for 30 minutes on ice in BM and washed thrice with PBS. Full-length recombinant S protein (Acrobio systems), and S1 subunit recombinant protein (a gift from Dr. Abraham Pinter) were diluted with BM to appropriate concentrations. Filtered Pseudotyped SARS-CoV-2 S was used immediately following filtration without further dilution. Pseudotyped SARS-CoV-2 S, or 1 µg of full-length recombinant S protein, or 1 µg of S1 subunit recombinant protein was added to designated wells of a 96-well V bottom plate. The plate was centrifuged at 600 × g for 30 minutes at 32°C, and subsequently incubated at 37°C at 5% CO_2_ for 1 hour. Cells were washed twice with PBS, stained with anti-S1 in FACS buffer (2% FBS in PBS) for 30 minutes on ice and washed thrice with PBS. Cells were then stained with goat anti-rabbit secondary antibody in FACS buffer for 30 minutes on ice, washed thrice with PBS, and analyzed by flow cytometry.

### Flow Cytometry Analysis

Cells were analyzed on a FACS LSRII or an LSR Fortessa or an BD Accuri C6 flow cytometer. PMT voltages were adjusted and compensation values were calculated before data collection. Data were acquired using FACS Diva software and analyzed using FlowJo software.

### CD107a Degranulation Assay

Degranulation assay was performed as described (26).

### Intracellular Cytokine Assay

Intracellular cytokine assay was performed as described previously (27).

### Cr^51^ Release Assay

To evaluate the direct cytotoxic activity of CAR-NK-92MI cells, Cr^51^ assay was performed as described previously (27). To evaluate the direct killing activity of primary CAR-NK cells, both expanded NK cells and CAR-NK cells were blocked with 1:200 anti-CD16 and 1:200 anti-NKG2D in FACS buffer for 30 minutes on ice prior to performing the Cr^51^ release assay as described.

### Statistical Analysis

Data were represented as means ± SEM. The statistical significance was determined using a two-tailed paired Student *t* test, a one-way ANOVA, where indicated. P < 0.05 was considered statistically significant.

## Results

### Generation and Characterization of S309-CAR-NK-92MI Cells

**Figure 1:**
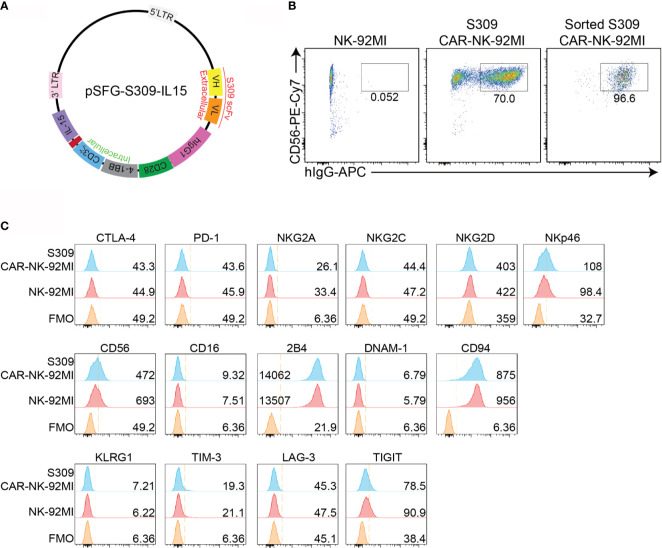
Generation of S309-CAR-NK-92MI cells. **(A)** Plasmid construct of S309-CAR. The SFG retroviral vector contains the S309 single chain antibody fragment (accession code 6WS6 on PDB), a human IgG1 CH2CH3 hinge region, a CD28 transmembrane region, followed by the co-stimulatory CD28, 4-1BB, and the intracellular domain of CD3ζ. **(B)** Determination of S309-CAR-NK expression by flow cytometry. S309-CAR cells were collected and stained with anti-CD56 and CAR F(ab)2 domain [IgG (H+L)] for flow cytometry. The cells were then sorted to achieve a homogenous population of high CAR expression. **(C)** Representative histogram showing the immunoprofiling of S309-CAR-NK-92MI cells by flow cytometry. S309-CAR-NK-92MI or the wildtype (WT) NK-92MI cells were stained with antibodies against different immunomodulatory receptors including CTLA-4, PD-1, NKG2A, NKG2C, NKG2D, NKp46, CD56, CD16, 2B4, DNAM-1, CD94, KLRG1, TIM-3, LAG-3, and TIGIT.

To develop an NK cell-based immunotherapy for a COVID-19 treatment, we cloned the scFv domain of S309 into an SFG retroviral vector (**Figure 1A**). After construction of S309-CAR, we successfully generated S309-CAR-NK cells in the human NK-92MI cell line (**Figure 1B**). Briefly, 293T cells were transfected with a combination of plasmids containing S309-CAR in the SFG backbone with retroviral helper plasmids RDF and PegPam3, as previously described (27). The SFG retrovirus particles were then used to transduce NK-92MI cells. After 4-5 days, NK-92MI and S309-CAR cells were stained with CD56 and human IgG (H+L). The CAR expression on NK-92MI cells was analyzed by flow cytometry. Around 70% of CD56^+^ S309-CAR^+^ NK-92MI cells were observed (**Figure 1B**). Then, the subsequent S309-CAR positive NK-92MI cells were sorted by flow cytometry to achieve high CAR expression levels (**Figures 1B** and **S1**). In summary, we have successfully established the surface expression of S309-CAR-NK-92MI cells.

To characterize S309-CAR-NK-92MI cells, we examined the expressions of several key immunoreceptors on S309-CAR-NK-92MI cells by flow cytometry. These receptors include TIGIT, LAG-3, TIM-3, KLRG1, CTLA-4, PD-1, CD69, NKG2C, CD94, DNAM-1, 2B4, NKG2D, NKp46, and CD16 (**Figure 1C**). Overall, the expressions of these activating and inhibitory receptors are comparable between parental NK-92MI and S309-CAR-NK-92MI cells, indicating the comparable characteristics of NK-92MI at pre- and post-transduction stages.

### S309-CAR-NK Cells Bind to the Immunogenic Receptor-Binding Domain (RBD) Domain of SARS-CoV-2 and Pseudotyped SARS-CoV-2 S Viral Particles

**Figure 2:**
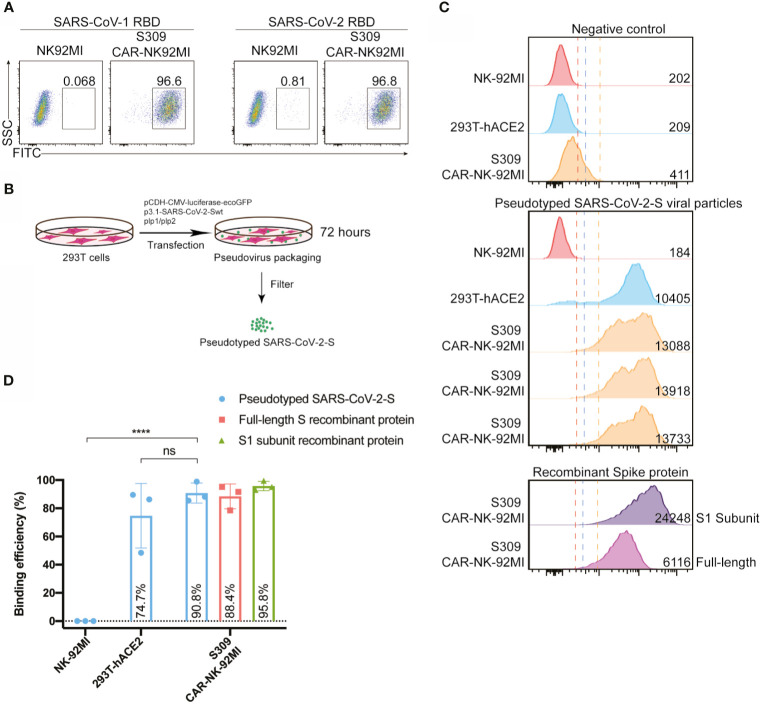
S309-CAR-NK-92MI cells bind to RBD domain of SARS-CoV-2 S protein and pseudotyped SARS-CoV-2 viral particles. **(A)** Representative dot plots showing the efficiency of S309-CAR binding to SARS-CoV-2-RBD. S309-CAR-NK-92MI or WT NK-92MI cells were incubated with the RBD recombinant protein of SARS-CoV-1 or SARS-CoV-2. **(B)** Generation of pseudotyped SARS-CoV-2 viral particles. 293T cells were transfected with various plasmids for 72 hours for the generation of pseudotyped SARS-CoV-2 viral particles. **(C)** Representative histogram showing S309-CAR-NK-92MI binds to the pseudotyped SARS-CoV-2 viral particles. S309-CAR-NK-92MI, parental NK-92MI, or 293T-hACE2 (positive control) cells were incubated with pseudotyped SARS-CoV-2 viral particles, S1 subunit, or full-length S recombinant protein at 37°C for 1 hour. The experimental sample was performed in triplicates with MFI = 13579 ± 251 (a.u.). **(D)** Quantitative data of the binding efficiency of S309-CAR-NK-92MI cells to pseudotyped SARS-CoV-2 viral particles. The experimental sample was performed in triplicates with binding efficiency of over 90%. Data represent means ± standard error of the mean (SEM) of three independent experiments represented by each dot. Non-parametric test was employed for panel **(D)**. ns p > 0.05, and ****p < 0.0001.

After successful establishment of S309-CAR-NK-92MI cells, we then assessed the binding ability of S309-CAR-NK cells to the RBD domain of SARS-CoV-2 S protein. Since S309 NAb was isolated from memory B cells of a SARS patient (23), we also included the recombinant His-RBD protein of SARS-CoV as a positive control. S309-CAR-NK-92MI cells and parental NK-92MI cells were incubated with the His-RBD of SARS-CoV or RBD-SARS-CoV-2 and the resulting complex was then recognized by anti-His and its corresponding fluorophore-conjugated-secondary antibody. Flow cytometry was employed to evaluate the binding efficiency of S309-CAR to the RBD of S protein from either SARS-CoV or SARS-CoV-2. Consistent with results from previous studies (28), S309 recognizes and strongly binds to the RBD of both SARS-CoV and SARS-CoV-2 (**Figure 2A**). We therefore conclude that S309-CAR-NK-92MI cells can specifically bind to the recombinant His-RBD protein of SARS-CoV and the RBD-SARS-CoV-2.

However, the partial RBD domain of SARS-CoV-2 S may not fully reflect the complexity of SARS-CoV-2 viral particles. We therefore evaluated the binding ability of S309-CAR-NK cells to pseudotyped SARS-CoV-2 S viral particles that we generated. Pseudotyped SARS-CoV-2 viral particles were produced by transfecting 293T cells with a combination of pCMV-luciferase-ecoGFP, pcDNA3.1-SARS-CoV-2 Spike, pLP1, and pLP2 plasmids. The supernatant containing pseudotyped SARS-CoV-2 viral particles were used for the pseudovirus binding assay (**Figure 2B**). To further confirm the presence of pseudotyped SARS-CoV-2 viral particles, we used the collected SARS-CoV-2 pseudovirus to infect 293T-hACE2 cells. After 48-72 hours of incubation, we observed the GFP expression of the infected 293T-hACE2 cells by EVOS florescence microscope (data not shown) and flow cytometry analysis **(** **Figure S2A** **).**

Previous studies show that the RBD of S glycoprotein binds to ACE2 and facilitates SARS-CoV-2 entry (29). Thus, we included 293T-hACE2 in our experiment as a positive control (**Figure 2C**). Full-length Spike and RBD-containing S1 subunit recombinant proteins were also included as additional control groups (**Figures 2C, D**). To evaluate the binding ability of S309-CAR-NK-92MI to the pseudotyped SARS-CoV-2 S virus, S309-CAR-NK-92MI, NK-92MI or 293T-hACE2 were incubated with SARS-CoV-2 S viral particles, S1 subunit, or full-length S recombinant protein. The complex can be recognized by anti-S1 subunit antibody and its corresponding fluorophore-conjugated secondary antibody. S309-CAR-NK-92MI cells were able to bind to the pseudotyped SARS-CoV-2 S viral particles with slightly lower binding efficiency than that of recombinant protein groups (**Figure 2C**), indicating the Spike protein expressed on the pseudotyped particles may be less accessible to S309-CAR-NK cells, compared to soluble recombinant Spike protein due to steric hindrance. Surprisingly, S309-CAR-NK cells bind to the pseudotyped SARS-CoV-2 viral particles with similar binding efficiency compared to that of 293T-hACE2 cells (**Figure 2D**). In summary, S309-CAR-NK-92MI cells can strongly and specifically bind to the RBD-containing S1 subunit recombinant protein and the full-length pseudotyped SARS-CoV-2 S viral particles.

### S309-CAR-NK Cells Can Be Activated by SARS-CoV-2 Spike Protein RBD Expressing Target Cells and Specifically Kill Their Susceptible Target Cells

**Figure 3:**
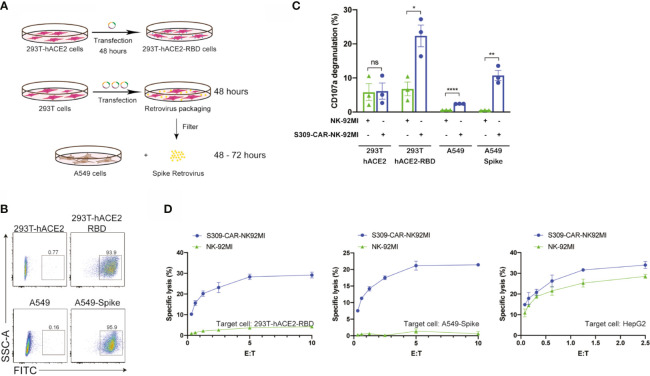
Increased CD107a surface expression and killing activity of S309-CAR-NK-92MI cells against 293T-hACE2-RBD and A549-Spike target cells. **(A)** Generation of transient 293T-hACE2-RBD and stable A549-Spike cell lines. 293T-hACE2 cells were transfected with RBD-containing plasmid for 48 hours. Transfected 293T-hACE2-RBD cells were then harvested. For the generation of A549-Spike, 293T cells were transfected with the retrovirus transfection system for 48 hours. The spike retrovirus was filtered and transduced into A549 cells for an additional 48-72 hours. **(B)** Representative dot plots showing the expressions of RBD or Spike in 293T-hACE2 or A549 cells, respectively. 293T-hACE2-RBD and A549-Spike cells were stained with anti-RBD and the expressions were confirmed by flow cytometry. The stable A549-Spike cell line was then sorted to achieve high levels of spike expression. **(C)** Quantitative data of CD107a surface expression assay of S309-CAR-NK against 293T-hACE2-RBD or A549-Spike cell lines. Briefly, S309-CAR-NK-92MI cells were cocultured with either 293T-hACE2-RBD cells, A549-Spike cells, stimulated with PMA/Ionomycin, or incubated alone for 2 hours at 37°C. Cells were then harvested and stained for CAR F(ab’)2 domain IgG (H+L) and CD107a. Each dot represents an experiment. Data represent means ± SEM from three independent experiments. **(D)** Representative 4-hour standard Cr^51^ release assay of S309-CAR-NK-92MI and parental NK-92MI cells against various target cell lines. 293T-hACE2-RBD, A549-Spike, and HepG2 cell lines were used as target cells for S309-CAR-NK-92MI and NK-92MI. Experimental groups were performed in triplicates. Error bars represent means ± SD. The experiment was repeated at least two times. Non-parametric test was used for panels **(C)**. ns p > 0.05, *p < 0.05, **p < 0.01, ****p < 0.0001.

After successful generation of S309-CAR-NK cells and demonstration of binding activities of recombinant His-RBD protein of SARS-CoVs (including SARS-CoV and SARS-CoV-2) and pseudotyped full length S viral particle derived from SARS-CoV-2, we further evaluated whether S309-CAR-NK cells can be activated by target cells expressing SARS-CoV-2 S protein. To test this, we generated two different cell lines expressing the RBD and S proteins using 293T-hACE2 and A549, respectively. For the generation of transient 293T-hACE2-RBD cells, we transfected an RBD encoding plasmid into 293T-hACE2 cells (a commonly used cell line for studying the SARS-CoV-2 virus) (**Figure 3A**). On average, the transfection efficiencies of RBD protein on 293T-hACE2 cells were greater than 90% as determined by flow cytometry (**Figure 3B**) and confocal microscopy (data not shown). For the generation of the stable A549-Spike cell line (**Table S2**), we transfected 293T cells with RDF, Pegpam3, and SARS-CoV-2 S in SFG backbone to produce SARS-CoV-2 S retrovirus that was subsequently transduced into A549 cells (**Figure 3A**). The S protein expression on A549 is weaker, compared to that of 293T cells (data not shown). The pre-sorting transduction efficiency on A549 cell line was around 70% verified by flow cytometry. Transduced A549-Spike cells were subsequently sorted to achieve homogeneously high expression levels of S protein compared with 293T-hACE2-RBD cells (**Figure 3B**).

Next, we examined the activation of S309-CAR-NK-92MI cells by 293T-hACE2-RBD or A549-Spike using the conventional CD107a assay (30). Expectedly, we observed a significant increase in the surface level expression of CD107a molecules on S309-CAR-NK-92MI cells after co-culturing with susceptible 293T-hACE2-RBD or A549-Spike compared to that of parental 293T-hACE2 or A549 cells. An increase in CD107a percentage (%) on S309-CAR-NK-92MI cells, compared to that of NK-92MI cells, was observed (**Figure 3C**). In conclusion, S309-CAR-NK-92MI cells can be activated by both 293T-hACE2-RBD and A549-Spike cells.

To evaluate the killing activity of S309-CAR-NK-92MI against SARS-CoV-2-protein-expressing target cells *in vitro*, we used the standard 4-hour Chromium-51 (Cr^51^) release assay (a gold standard assay to evaluate the cytotoxicity of immune cells) (31). The data show that S309-CAR-NK-92MI cells effectively kill both 293T-hACE2-RBD and A549-Spike cells by *in vitro* Cr^51^ release assay (**Figure 3D** **)**. We also used an irrelevant target cell line, HepG2 (human hepatoma cell line) as a negative control, to confirm the specificity of our S309-CAR-NK. As expected, we did not observe a significant difference in the killing activity of S309-CAR-NK-92MI compared to that of wild-type NK-92MI cells (**Figure 3D**). Here, by using three different cell lines, we demonstrated the S309-CAR-NK-92MI cells can specifically target and kill SARS-CoV-2-protein-expressing target cells.

### Expanded Primary S309-CAR-NK Cells Can Specifically Kill SARS-CoV-2-Protein-Expressing Target Cells

**Figure 4:**
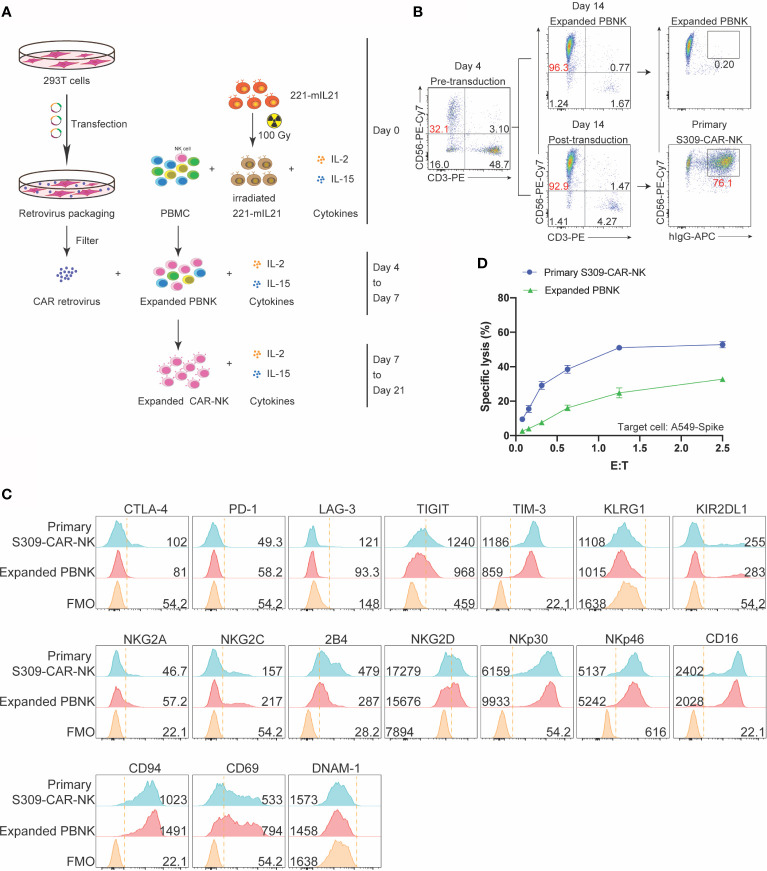
Increased killing activity of expanded S309-CAR-NK^primary^ against A549 Spike cell line. **(A)** Schematic representation of human S309-CAR-NK^primary^ expansion system. Briefly, irradiated (100 Gy) 221-mIL21 feeder cells were cocultured with PBMC supplemented with IL-2 and IL-15 on Day 0. In parallel, 293T cells were transfected with the retrovirus packaging system to produce S309-CAR retrovirus that were then transduced into the expanded PBNK cells in the presence of IL-2 and IL-15. S309-CAR-NK^primary^ cells were harvested on Day 7 and continued expansion for 21 days. **(B)** Representative dot plots of expanded primary NK cells and S309-CAR-NK^primary^. The purity of NK cells and the expression of CAR were monitored every 3-4 days. **(C)** Representative histogram of immunophenotyping of S309-CAR-NK^primary^ cells using flow cytometry. Antibodies against various immunomodulatory receptors including CTLA-4, PD1, NKG2A, CD56, CD16, 2B4, NKG2C, NKG2D, NKp30, NKp46, DNAM-1, CD69, CD94, TIGIT, KLRG1, KIR2DL1, TIM-3, and LAG-3 were used to stain both primary NK cells and S309-CAR-NK^primary^. **(D)** Representative quantitative data of cytotoxic activity of S309-CAR-NK^primary^ against A549-Spike. Briefly, expanded human S309-CAR-NK^primary^ cells were blocked with anti-CD16 for 30 minutes and then anti-NKG2D for 30 minutes on ice. The target cells were labeled with Cr^51^ for 2 hours prior to coculturing with S309-CAR-NK^primary^ cells for an additional 4 hours. Representative data are shown in panel **(D)**.

After evaluating the killing function of S309-CAR-NK-92MI cell line, we then examined whether the expanded S309-CAR-modified primary NK cells (henceforward, S309-CAR-NK^primary^) isolated from human peripheral blood also have similar killing function against SARS-CoV-2-protein-expressing cells, given the natural malignancy of NK-92MI cell line (17). To expand human primary NK cells from peripheral blood (hereinafter PBNK), we isolated peripheral blood mononuclear (PBMCs) from buffy coats from at least four healthy donors and cocultured them with 100-Gy irradiated 221-mIL21 feeder cells supplemented with 200 U/mL IL-2 and 5 ng/mL IL-15, as described (26). In parallel, 293T cells were transfected with S309-CAR in the SFG backbone, RDF, and PegPam3 plasmids, as previously described (27). The SFG retrovirus particles were used to transduce expanded PBNK cells at Day 4 (**Figure 4A**). After 48 hours, primary S309-CAR-NK cells were transferred to a G-Rex plate for continued culturing for 21 days. The NK cell purity and CAR expression were determined using flow cytometry by staining both PBNK and S309-CAR-NK^primary^ cells with anti-CD56, anti-CD3 and anti-human IgG (H+L). On average, the NK cell purity is around 90% with approximately 80% CAR transduction efficiencies for the S309-CAR-NK^primary^ (**Figure 4B**).

To immunophenotype the expanded S309-CAR-NK^primary^ cells, we stained both expanded PBNK and S309-CAR-NK^primary^ cells for various important activating and inhibitory receptors by which the expressions were determined by flow cytometry. The inhibitory receptors include CTLA4, PD-1, NKG2A, TIGIT, KLRG1, TIM3, and LAG3. The activating receptors include CD16, 2B4, NKG2C NKG2D, DNAM-1, NKp30, and NKp46 (**Figure 4C**). We observed that surface 2B4, NKG2D, TIM-3, and CD16 on S309-CAR-NK^primary^ increased slightly, suggesting that S309-CAR-NK^primary^ cells are better regulated compared to that of un-transduced expanded PBNK cells. In general, the expressions of these immunomodulatory receptors are comparable between expanded PBNK and S309-CAR-NK^primary^ cells.

Similar to S309-CAR-NK-92MI, we also used the 4-hour Cr^51^ release assay to evaluate the killing function of expanded S309-CAR-NK^primary^. The data show that S309-CAR-NK^primary^ cells effectively kill A549-Spike cells, compared to expanded PBNK cells (**Figure 4D** **)**. In summary, we demonstrated the expanded S309-CAR-NK^primary^ cells can also kill the SARS-CoV-2-protein-expressing target cells, indicating S309-CAR-NK^primary^ could potentially become a promising COVID-19 treatment.

### S309-CAR-NK Cells Have Higher Killing Activities Than CR3022-CAR-NK Cells Against SAR-CoV-2-Protein-Expressing Cells

**Figure 5:**
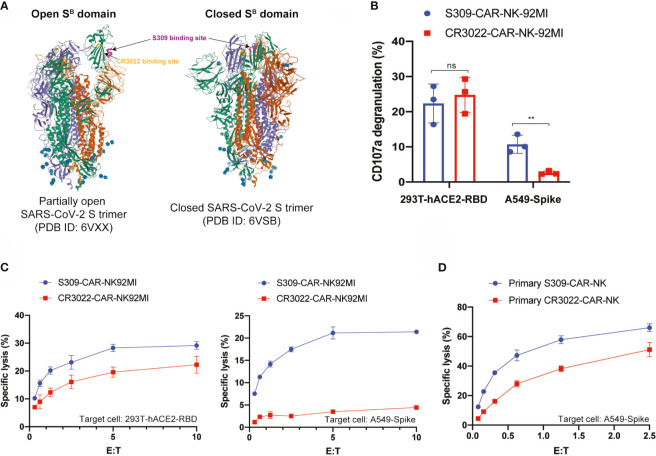
S309-CAR-NK cells are superior to CR3022-CAR-NK cells. **(A)** Diagram of S309 and CR3022 NAbs binding to different epitopes of the SARS-CoV-2 S protein. Both open and closed conformation states of SARS-CoV-2 S protein are shown. S309 binding site is indicated in magenta and CR3022 binding site is indicated in yellow. **(B)** Quantitative data of CD107a surface expression of both S309-CAR-NK-92MI and CR3022-CAR-NK-92MI. Both transient 293T-hACE2-RBD and stable A549-Spike cell lines were used as target cells. Error bars represent SEM from at least two independent experiments, each dot representing an experiment. **(C)** Comparison of killing activity of S309-CAR and CR3022-CAR using the 4-hour Cr^51^ release assay. Effector cells were cocultured with Cr^51^-labeled target cells at 37°C for 4 hours. The assay was repeated for at least two times per target cell line. Representative data from one experiment is shown. Error bars represent means ± SD. **(D)** Expanded S309-CAR-NK^primary^ has increased killing activity against A549-Spike cells compared to primary CR3022-CAR-NK^primary^. Effector cells were blocked with anti-CD16 and anti-NKG2D prior to coculturing with A549-Spike target cells for 4 hours at 37°C. S309-CAR-NK^primary^ and CR3022-CAR-NK^primary^ cells were expanded from three different donors. Representative data from one heathy donor are shown. Error bars represent means ± SD. Non-parametric test was employed for panel **(B)**. ns p > 0.05 and **p < 0.01.

CR3022-CAR-NK-92MI cell line was previously generated (24). Previous studies showed that S309 NAb recognizes both open and closed conformations of the SARS-CoV-2 S trimer, however, CR3022 can only bind to the open state (**Figure 5A**) (23, 32). To further determine the functionalities of S309-CAR-NK cells *in vitro*, we compared the activation levels of S309-CAR-NK-92MI and CR3022-CAR-NK-92MI cells when cocultured with susceptible 293T-hACE2-RBD or A549-Spike target cells. To ensure that the difference in S309-CAR-NK-92MI or CR3022-CAR-NK-92MI activation is not the direct consequence of the differences in CAR expressions, we first compared the expressions of S309-CAR and CR3022-CAR by flow cytometry (**Figure S1**). We subsequently performed a degranulation assay to detect the activation of S309-CAR-NK-92MI or CR3022-CAR-NK-92MI cells against target cells expressing RBD or Spike protein. As expected, we did not observe a significant difference in the degranulation using CD107a assay against the 293T-hACE2-RBD target cells, indicating both CR3022- and S309-CAR can bind with RBD protein in a comparable manner. However, the expression levels of surface CD107a on S309-CAR-NK cells were significantly higher when cocultured with A549-Spike, compared to that of CR3022-CAR-NK cells (**Figure 5B**). Altogether, these data suggest that the conformation of the SARS-CoV-2 Spike trimers play a critical role in CAR recognition and binding ability and superior activation capabilities by S309-CAR molecules, which is measured by standard CD107a degranulation assays.

To further evaluate the function of S309-CAR-NK-92MI cells, we compared their killing activities to the CR3022-CAR-NK-92MI cells. Both 293T-hACE2-RBD and A549-Spike cells were used as susceptible target cells. Consistent with the results in **Figure 5B**, we also observed a significant decrease in the killing activity of CR3022-CAR-NK cells when A549-Spike cells were the susceptible target cell line in a 4-hour Cr^51^ release assay (**Figure 5C**). Considering NK-92MI is a cell line and may not fully reflect the true functions of primary CAR-NK cells (17), we also generated S309-CAR-NK^primary^ and CR3022-CAR-NK^primary^ using primary NK cells expanded from at least four different healthy donors. We observed that S309-CAR-NK^primary^ cells have a higher trend of killing activities compared to that of CR3022-CAR-NK^primary^ (**Figure 5D**). Our data indicate that S309-CAR-NK may be superior to CR3022-CAR-NK and may have a better potential in targeting and killing SARS-CoV-2 S-protein-expressing cells *in vitro*.

### S309-CAR-NK^primary^ Cells Can Also Target SARS-CoV-2 D614G, K484, and Y501 Variants

**Figure 6:**
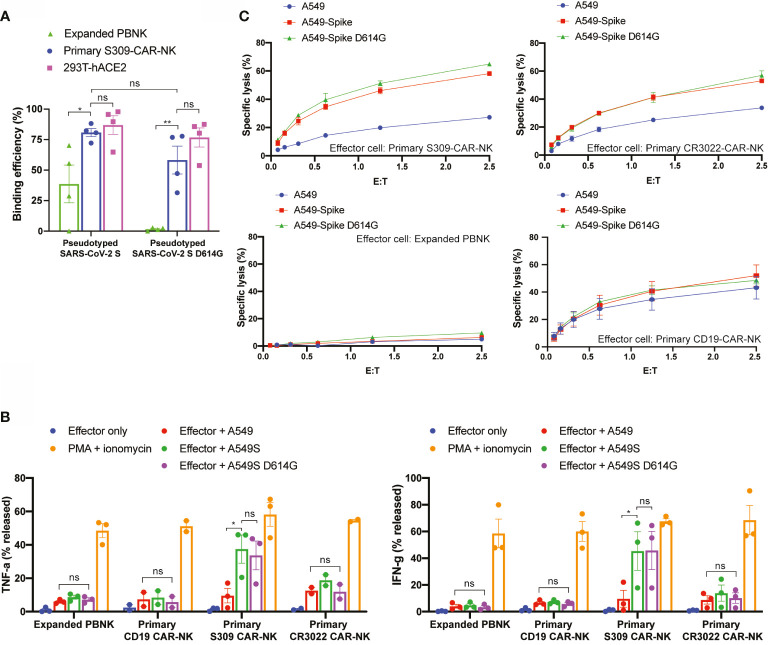
S309-CAR-NK^primary^ cells can also target SARS-CoV-2 D614G variant. **(A)** Quantitative data of the binding efficiency of S309-CAR-NK^primary^ to pseudotyped SARS-CoV-2 and D614G variant viral particles. **(B)** Intracellular cytokine productions of various effector cells. Briefly, effector cells were cocultured with A549, A549-Spike, or A549-Spike D614G for 4 hours at 37°C. Cells were then stained for surface markers and permeabilized prior to staining for intracellular cytokines. Data were acquired by flow cytometry and the values were normalized with the PMA + ionomycin group (positive control). Data were pooled from at least two independent experiments. **(C)** Expanded S309-CAR-NK^primary^ has increased killing activity against A549-Spike and A549-Spike D614G cells compared to CR3022-CAR-NK^primary^. Effector cells were blocked with anti-CD16 and anti-NKG2D prior to coculturing with A549-Spike or A549-Spike D614G target cells for 4 hours at 37°C. Primary NK cells and CAR-NK^primary^ cells were expanded from at least four donors. Representative data from one donor is shown. Samples were performed in triplicates. Error bars represent means ± SD. Non-parametric test was employed for panels **(A, B)**. ns p > 0.05 and *p < 0.05.

Next, we evaluated the ability by which S309-CAR-NK^primary^ cells bind to pseudotyped SARS-CoV-2 D614G variant by flow cytometry. As expected, S309-CAR-NK^primary^ cells are unaffected by the G614 mutation and have the ability to bind to pseudotyped SARS-CoV-2 G614 variant with similar binding efficiency compared to the SARS-CoV-2 D614 **(** **Figure 6A** **)**. However, the binding of pseudotyped SARS-CoV-2 viral particles, both G614 and D614, did not result in activation of S309-CAR-NK^primary^ cells confirmed by CD107a degranulation assay (**Figure S3**). To ensure the functions of S309-CAR-NK^primary^ are not altered by the G614 mutation, we also generated A549-Spike D614G cell line. Site-directed mutagenesis was performed to mutagenize SFG-SARS-CoV-2 S plasmid (**Table S2**). Subsequently, we generated A549-Spike D614G cell line using the retrovirus system, which 293T cells were transduced with a combination of RDF, Pegpam3, and SARS-CoV-2 S D614G in SFG backbone. We then performed an intracellular cytokine assay to quantify the intracellular productions of TNF-α and IFN-γ of S309-CAR-NK^primary^ cells **(** **Figure 6B** **)**. In addition to using expanded PBNK, CD19-CAR-NK cells (specific for the treatment of B-acute lymphoblastic leukemia) (20) were also included to show the specificity of S309-CAR-NK^primary^ against target cells expressing wild-type S protein or the D614G variant. Consistent with our previous result in **Figure 5D**, S309-CAR-NK^primary^ produce higher amount of intracellular TNF-α and IFN-γ compared to that of CR3022-CAR-NK^primary^ when cocultured with A549-Spike target cells. We also demonstrated that the NK cell population expressing S309-CAR is the major source of TNF-α and IFN-γ productions, but not the T cell population (**Figure S4**). More importantly, the intracellular cytokine productions of S309-CAR-NK^primary^ cells when cocultured with A549-Spike D614G target cells were similar to that of A549-Spike, indicating S309-CAR-NK^primary^ cells can also target and kill cells infected by the SARS-CoV-2 D614G variant. Furthermore, we also showed that S309-CAR-NK^primary^ can bind to other variants of pseudotyped SARS-CoV-2 viral particles, including the E484K and N501Y mutations (**Figure S5A**).

Next, we used the Cr^51^ release assay to further determine the killing activities of S309-CAR-NK^primary^ cells against A549-Spike D614G target cells. Consistently, S309-CAR-NK^primary^ cells can also specifically kill A549-Spike D614G cells, indicating that S309-CAR-NK^primary^ cells are unaffected to the G614 variant **(** **Figure 6C** **)**. Additionally, S309-CAR-NK^primary^ cells are able to bind various pseudotyped SARS-CoV-2 variants in addition to directly killing target cells expressing S protein exhibiting mutations including the G614, K484, and Y501 **(** **Figures 6C** and **S5B** **)**. In summary, S309-CAR-NK^primary^ cells can specifically recognize and target the emerging SARS-CoV-2 variants including the G614, K484, and Y501 *in vitro*.

## Discussion

Recent clinical trials of CAR-modified NK cell-based immunotherapies showed promising results in treating cancers and infectious diseases (17). Compared to that of CAR-T cell-based therapy, CAR-NK cell-based immunotherapy has been shown to be more advantageous due to better clinical safety profiles and the allogeneic nature of CAR-NK. The unique recognition of tumor cells or virally infected cells by NK cells *via* the major histocompatibility complex (MHC) nonrestricted manner is crucial for NK cell-mediated cytotoxicity without significant side effects in the clinical setting. Another advantage of using CAR-NK cell-based therapy is that NK cells produce minimal proinflammatory cytokine IL-6, which has been shown to significantly contribute to the cytokine release syndrome (CRS) that is caused by CAR-T immunotherapy or observed in severe COVID-19 patients (33, 34). However, one of the foremost challenges of using primary NK cells for immunotherapy is obtaining an adequate number of functional, non-exhaustive NK cells from peripheral blood or cord blood during expansion. Therefore, most of the current CAR-NK immunotherapies in clinical trials utilize NK-92 cell lines as they are easily manufactured for “off-the-shelf” purposes (35). The superior and optimized human primary NK cell expansion technology has been recently developed to improve the scalability of future CAR-NK products (26). However, whether CAR-NK cells play a role in protecting patients with COVID-19 remains unsolved. The combination of CAR-NK with newly developed CAR-NK expansion technologies will make it possible to interrogate this important question.

In this study, we proposed the use of S309-CAR-NK cells for the treatment of COVID-19 for various reasons. Firstly, S309 is a unique antibody by which it targets one of the most highly conserved epitopes in the RBD of SARS-CoV-2 and related viruses, possessing high binding affinities and neutralization potencies for both SARS-CoV and SARS-CoV-2 (23). Our results suggest that this approach would retain activity against the Spike gene variants that are rapidly spreading around the globe, which may result in the development of escape mutants to the typical NAbs in convalescent and vaccine sera. This high level of conservation suggests that S309 antibody, and the CAR-NK cells derived from this antibody, may also possess protective activity against new SARS-CoV isolates that may emerge in the future. Secondly, accumulating evidence shows that NK cells are involved in the control of various viral infections (17, 20). As innate immune cells, NK cells can rapidly respond to viral infections by secreting IFN- and TNF- cytokines to upregulate the defense mechanism. NK cells can also directly recognize and target viral-infected cells *via* antibody-dependent cellular cytotoxicity (ADCC). Thirdly, the half-life of monoclonal antibody therapeutics is approximately 14-21 days; however, expression of the antibody scFv portion on NK cells can prolong the protection by increasing the persistence of NK cells by the addition of critical NK cell survival cytokines (e.g., IL-15) *in vivo*. Thus, the functions of S309-CAR-NK cells in controlling COVID-19 progression can be classified into two aspects: 1) The direct binding of S309-CAR molecules to free SARS-CoV-2 virus particles; 2) The activation of CAR-NK cells *via* the binding of S309-CAR or NKG2D or other natural receptors exerts natural cytotoxicity to directly kill SARS-CoV-2-infected cells. Given these reasons, it is essential to design genetically modified NK cells that specifically target SARS-CoV-2 infected cells to control COVID-19 disease progression during the appropriate treatment window following infection.

Here, we show the successful generation of S309-CAR-NK cells using both the NK-92MI cell line and primary NK cells expanded from human peripheral blood. We consistently expanded primary NK cells to around 90% NK cell purity from at least four healthy donors in addition to achieving high levels of CAR transduction efficiencies (approximately 80%) and showing the functionalities of S309-CAR-NK^primary^ cells against A549 target cell line expressing Spike protein bearing G614, K484, or Y501 mutation. We showed that S309-CAR-NK cells have the ability to efficiently bind to different variants of SARS-CoV-2. However, the binding of S309-CAR-NK^primary^ cells to the pseudotyped SARS-CoV-2 viral particles does not result in S309-CAR-NK^primary^ cells activation. Therefore, we speculate that the activation of S309-CAR-NK^primary^ cells may require a synergistic effect from both the S309-CAR and another receptor, e.g., a costimulatory receptor or an activating receptor. We also demonstrated the cytotoxic functions of S309-CAR-NK against different target cells expressing RBD, Spike D614, and Spike G614 in various *in vitro* assays. We observed that NK cells are the major source of TNF-α and IFN-γ release and not T cells *in vitro*, indicating that S309-CAR-NK^primary^ cells are the major cell type that directly target SARS-CoV-2-infected cells.

Interestingly, we observed the superior cytotoxic functions for S309-CAR-NK, compared to that of CR3022-CAR-NK cells against A549-Spike target cells. A plausible explanation is the different epitopes and affinities of these two NAbs to SARS-CoV-2 S glycoprotein. CR3022 binds to a cryptic epitope that is only accessible when at least two out of the three S^B^ domains of S glycoprotein trimers are in the open conformation (32), while S309 binds to the SARS-CoV-2 S^B^ domain and to the ectodomain trimer of the S glycoprotein (23). Furthermore, S309 NAb recognizes the N343 glycan that is located outside of the ACE2-binding site, and the acquired mutations of the emerging SARS-CoV-2 variants, including the N501Y, E484K, or K418T, are not within the S309 epitope (36). This is reflected in neutralizing activity of S309, but not CR3022 for SARS-CoV-2, and this difference in the specific binding domains of CR3022 and S309 could explain the superior killing activities of S309-CAR-NK cells. Collectively, these preliminary data support the therapeutic potential of using S309-CAR-NK cells as a treatment for severe COVID-19 patients.

However, several limitations in the current study need to be addressed as follows:

(1) The pseudotyped SARS-CoV-2 S viral particles or A549 cell line expressing the Spike protein used in our experiments may not fully recapitulate the authentic SARS-CoV-2 virus or SARS-CoV-2-infetced host cells. It will be more informative if the authentic SARS-CoV-2 virus or SARS-CoV-2-infected cells was used for *in vitro* experiments, however, this requires BSL3 access which is currently limited.
(2) The current studies provide inadequate mechanistic insights showing how S309-CAR-NK cells target SARS-CoV-2 virus or SARS-CoV-2 S expressing cells. Currently, we are testing the efficacy of S309-CAR-NK cells in animal studies, and our current study supports the potential efficacy of S309-CAR-NK cells and the safety assessment of S309-CAR-NK cells *in vivo*.
(3) The use of S309-CAR-NK^primary^ cells for the treatment of COVID-19 may also possess three potential logistics limitations including limited accessibility, high costs, and complicated manufacturing or production, which represent the common challenges in the field of cell therapy. However, the development of CAR-NK cells is evolving at an unprecedented speed, it is highly expected that these limitations will be resolved in the near future. The potential ‘off-the-shelf’ CAR-NK products by using the NK-92 cell line will also potentiate the use of S309-CAR-NK cells during the short treatment windows of SARS-CoV-2 infection (37). Our lab has also established a superior NK cell expansion technology that improves the NK cell purity and the NK cell expansion rate with enhanced *in vivo* functions, which will eventually reduce the costs of CAR-NK cell generation (26, 38). Overall, combining the ‘off-the-shelf’ potential and the optimized *ex vivo* expansion technology, we expect that S309-CAR-NK cells will become an invaluable clinical armamentarium in fighting against emerging SARS-CoV-2 variants.

Future studies using wild-type SARS-CoV-2 virus in pre-clinical animal models are needed to test the efficacy and toxicity of S309-CAR-NK cells *in vivo*. Future plans include characterization of the CAR-NK^primary^ toxicity in the preclinical animal models and clinical trials.

The development of this novel CAR-NK cell therapy for the treatment of severe COVID-19 patients with maximal efficacy and minimal toxicity will be required to reduce patient risk and enhance the clinical benefit of these expensive and time-intensive therapies in the acute care setting. As S309-CAR-NK^primary^ cells can directly target cells expressing Spike protein bearing various mutations from the emerging SARS-CoV-2 variants, we speculate that the CAR-NK approach may provide an additional clinical immunotherapeutic to help combat the future SARS-CoV-2-related pandemics. This work also pioneers the use of S309-CAR-NK cells to treat SARS-CoV-2 infected patients and will lead to the development of novel immunotherapeutic strategies for patients with immunocompromised conditions. These patients include: 1) older adults (particularly over 70-year-old with immune-compromising conditions); 2) those with comorbidities or chronic conditions such as cancer, heart disease, pre-existing lung disease, diabetes, malnutrition, and certain genetic disorders; 3) those on specific medications or treatments such as steroids, chemotherapy, radiation therapy, stem cell therapy, bone marrow or organ transplant, who may not create an effective immune protection elicited by vaccines.

Furthermore, this study will expedite preclinical studies and a potential clinical application of “off-the-shelf” CAR-NK products for COVID-19 treatment. This work will also pioneer the use of a cancer immunotherapy in treating infectious diseases, broaden the use of CAR-NK cells, and initiate the development of ‘off-the-shelf’ CAR-NK products in the near future.

## Data Availability Statement

The raw data supporting the conclusions of this article will be made available by the authors, without undue reservation.

## Author Contributions

MM, SB, and DL designed the study and wrote the manuscript, other authors assisted with experiments and manuscript preparation. DL conceived and supervised the study. All authors contributed to the article and approved the submitted version.

## Funding

This work was supported in part from HL125018 (DL), AI124769 (DL), AI129594 (DL), AI130197 (DL), and Rutgers-Health Advance Funding (NIH REACH program), U01HL150852 (R. Panettieri, S. Libutti, and R. Pasqualini), S10OD025182 (DL), and Rutgers University-New Jersey Medical School Startup funding for DL Laboratory.

## Conflict of Interest

The authors declare that the research was conducted in the absence of any commercial or financial relationships that could be construed as a potential conflict of interest.

## Publisher’s Note

All claims expressed in this article are solely those of the authors and do not necessarily represent those of their affiliated organizations, or those of the publisher, the editors and the reviewers. Any product that may be evaluated in this article, or claim that may be made by its manufacturer, is not guaranteed or endorsed by the publisher.

## Supplementary Material

Supplementary Figure 1 CAR expression of S309 and CR3022 in NK-92MI cell line confirmed by flow cytometry. **(A)** Flow cytometric analysis of S309-CAR-NK-92MI and CR3022-CAR-NK-92MI. Parental NK-92MI was used as a control. Cells were stained for goat anti-human IgG (H+L). The CAR expression is comparable between S309-CAR and CR3022-CAR, at 88.7% and 95.4%, respectively **(B)** Offset flow cytometric profile of S309-CAR-NK-92MI and CR3022-CAR-NK-92MI. The CAR expression is between 10^4^ and 10^5^ mean fluorescence intensity (MFI) for both S309-CAR and CR3022-CAR. Data are representative of at least two independent experiments.

Supplementary Figure 2 Confirmation of pseudotyped SARS-CoV-2 D614 and SARS-CoV-2 G614 viral particles production. **(A)** Briefly, produced SARS-CoV-2 pseudovirus was used to infect 293T-hACE2 cells. After 48-72 hours, 293T-hACE2 cells were collected to detect SARS-CoV-2 S viral particles by flow cytometry. Intracellular or surface staining was performed with anti-RBD in permeabilization wash buffer or FACS buffer, respectively, for 30 minutes. Cells were then washed and stained for goat anti-rabbit AF488 prior to flow cytometry analysis. **(B)** Confirmation of pseudotyped SARS-CoV-2 S variants. 293T-hACE2 cell line was cocultured with media only or various pseudotyped SARS-CoV-2 S variants for 2 hours at 37°C. Cells were subsequently stained extracellularly for Spike with rabbit anti-RBD followed by secondary anti-rabbit prior to flow cytometry.

Supplementary Figure 3 S309-CAR-NK^primary^ cells is not activated upon pseudotyped SARS-CoV-2 viral particle binding. **(A)** Degranulation CD107a of S309-CAR-NK^primary^ cells upon pseudotyped SARS-CoV-2 S viral particle binding. Briefly, either expanded PBNK or S309-CAR-NK^primary^ cells were cocultured with media only, pseudotyped SARS-CoV-2 S, or pseudotyped SARS-CoV-2 S D614G for 2 hours at 37°C, where A549 or A549-Spike or A549-Spike D614G cells were used as positive controls, respectively. Cells were then collected and stained for anti-CD56, anti-CD3, anti-hIgG F(ab’)_2_, anti-CD107a, and anti-Spike subunit 1 (anti-S1) followed by flow cytometry. **(B)** Confirmation of pseudotyped SARS-CoV-2 viral particle binding to S309-CAR-NK^primary^ cells. Representative data are shown. Error bars represent means ± SD. The experiment was performed two times.

Supplementary Figure 4 Representative dot plots showing the INF-γ or TNF-α release of CD56^+^ NK cells or CD56^+^ CD3^+^ cells or CD3^+^ T cells. Expanded PBNK or primary S309-CAR-NK^primary^ cells were cocultured with A549 Spike target cells for 2 hours at 37°C in the presence of GolgiStop. Cells were subsequently stained for anti-CD56, anti-CD3, and anti-hIgG (H+L)(Fab’)_2_ followed by cell permeabilization and stained for anti-IFN-γ or anti-TNF-α. Flow cytometry was performed to determine the intracellular cytokine production.

Supplementary Figure 5 S309-CAR-NK^primary^ cells are activated by other SARS-CoV-2 variants. **(A)** S309-CAR-NK^primary^ cells bind to pseudotyped SARS-CoV-2 viral particles bearing N501Y or E484K. Briefly, the pcDNA3.1-SARS-CoV-2 S plasmid was mutagenized using the primers listed in **Table S1**. The pseudotyped SARS-CoV-2 viral particles were generated using the described method. S309-CAR-NK^primary^ or expanded PBNK or 293T-hACE2 cells were incubated with the pseudotyped SARS-CoV-2 S viral particles containing D614G, or E484K, or N501Y, and wildtype (WT) was used as a positive control, for 2 hours at 37°C. Cells were subsequently stained extracellularly for Spike with rabbit anti-RBD followed by secondary anti-rabbit prior to flow cytometry. S309-CAR-NK^primary^ and expanded PBNK cells were expanded from one donor. Data were pooled from three independent experiments. **(B)** S309-CAR-NK^primary^ cells directly kill A549 cells expressing Spike bearing E484K or N501Y mutation. Expanded PBNK or S309-CAR-NK^primary^ cells were cocultured with A549, A549-Spike E484K, or A549-Spike N501Y for 4 hours at 37°C. Samples were performed in triplicates with means ± SD. Non-parametric test was employed for panel **(A)**. ns p > 0.05 and * p < 0.05.

Supplementary Figure 6 Chromium release assay of expanded NK cells and CAR-NK cells against A549-Spike or A549-Spike D614G. Peripheral blood mononuclear cells were isolated from the buffy coats received from New York Blood Center as described in the methods section. Primary NK cells were expanded and transduced using the retrovirus packaging system to generate S309-CAR-NK^primary^ or CR3022-CAR-NK^primary^ cells from three different healthy donors. Chromium release assay was performed by coculturing effector cells with A549-Spike or A549-Spike D614G target cells at different effector: target (E/T) ratios for 4 hours at 37°C. Representative data from one experiment per donor are shown. Error bars represent means ± SD.
