# Supplementary figures and images for: CAR-NK Cells Effectively Target SARS-CoV-2-Spike-Expressing Cell Lines In Vitro
Source: Front Immunol. 2021 Jul 23;12:652223. doi: 10.3389/fimmu.2021.652223 (PMC8343231; doi:10.3389/fimmu.2021.652223)

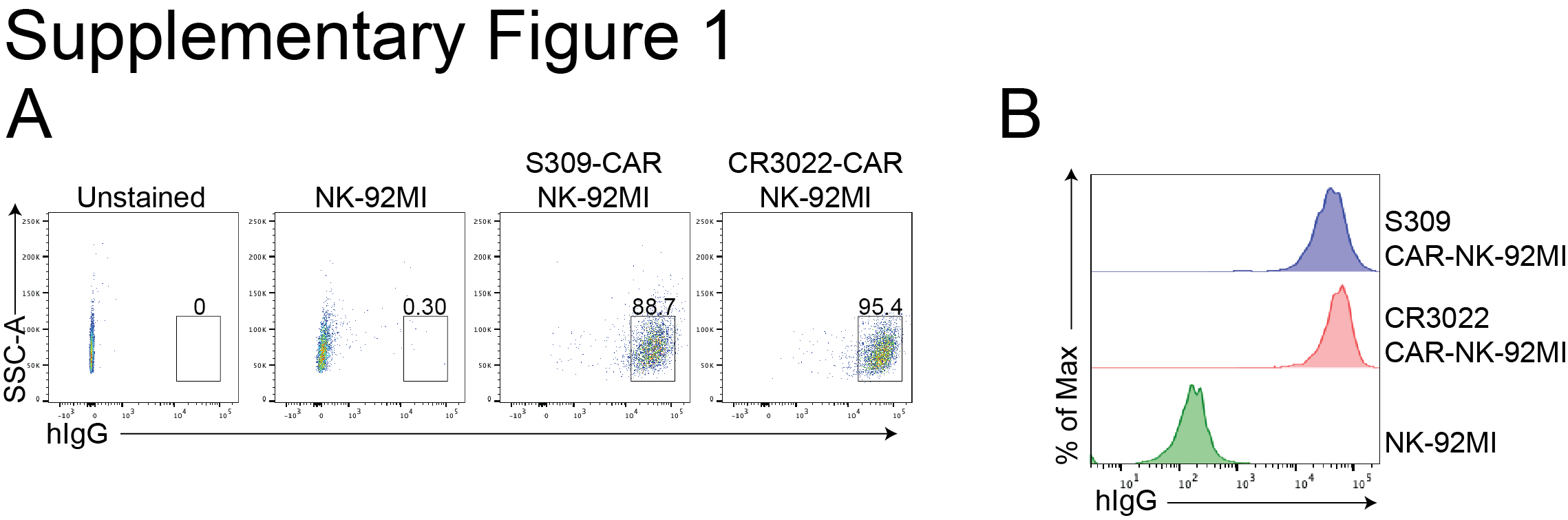

Supplement: Supplementary Figure 1 — CAR expression of S309 and CR3022 in NK-92MI cell line confirmed by flow cytometry. (A) Flow cytometric analysis of S309-CAR-NK-92MI and CR3022-CAR-NK-92MI. Parental NK-92MI was used as a control. Cells were stained for goat anti-human IgG (H+L). The CAR expression is comparable between S309-CAR and CR3022-CAR, at 88.7% and 95.4%, respectively (B) Offset flow cytometric profile of S309-CAR-NK-92MI and CR3022-CAR-NK-92MI. The CAR expression is between 104 and 105 mean fluorescence intensity (MFI) for both S309-CAR and CR3022-CAR. Data are representative of at least two independent experiments. [file Image_1.tif]

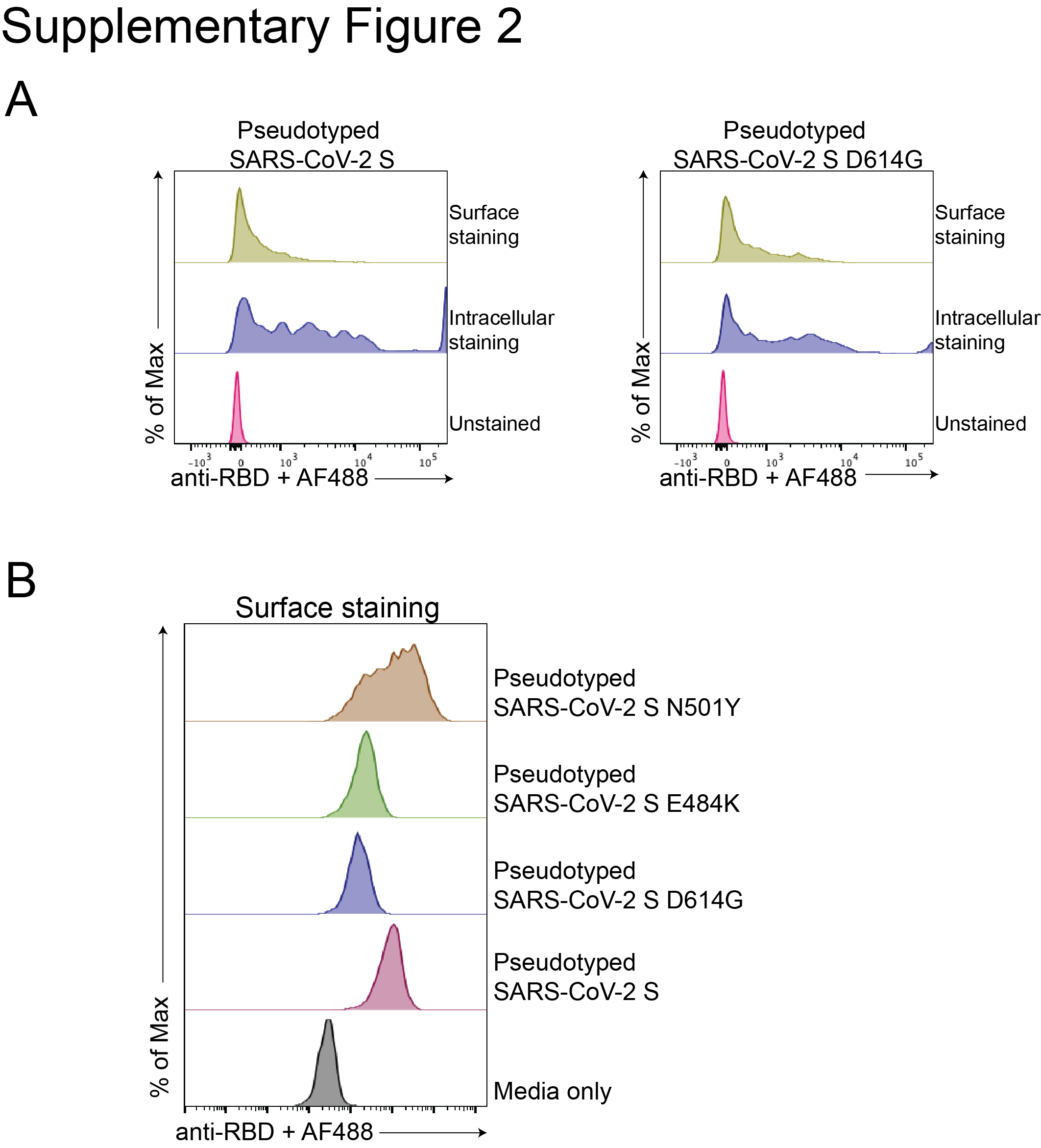

Supplement: Supplementary Figure 2 — Confirmation of pseudotyped SARS-CoV-2 D614 and SARS-CoV-2 G614 viral particles production. (A) Briefly, produced SARS-CoV-2 pseudovirus was used to infect 293T-hACE2 cells. After 48-72 hours, 293T-hACE2 cells were collected to detect SARS-CoV-2 S viral particles by flow cytometry. Intracellular or surface staining was performed with anti-RBD in permeabilization wash buffer or FACS buffer, respectively, for 30 minutes. Cells were then washed and stained for goat anti-rabbit AF488 prior to flow cytometry analysis. (B) Confirmation of pseudotyped SARS-CoV-2 S variants. 293T-hACE2 cell line was cocultured with media only or various pseudotyped SARS-CoV-2 S variants for 2 hours at 37°C. Cells were subsequently stained extracellularly for Spike with rabbit anti-RBD followed by secondary anti-rabbit prior to flow cytometry. [file Image_2.tif]

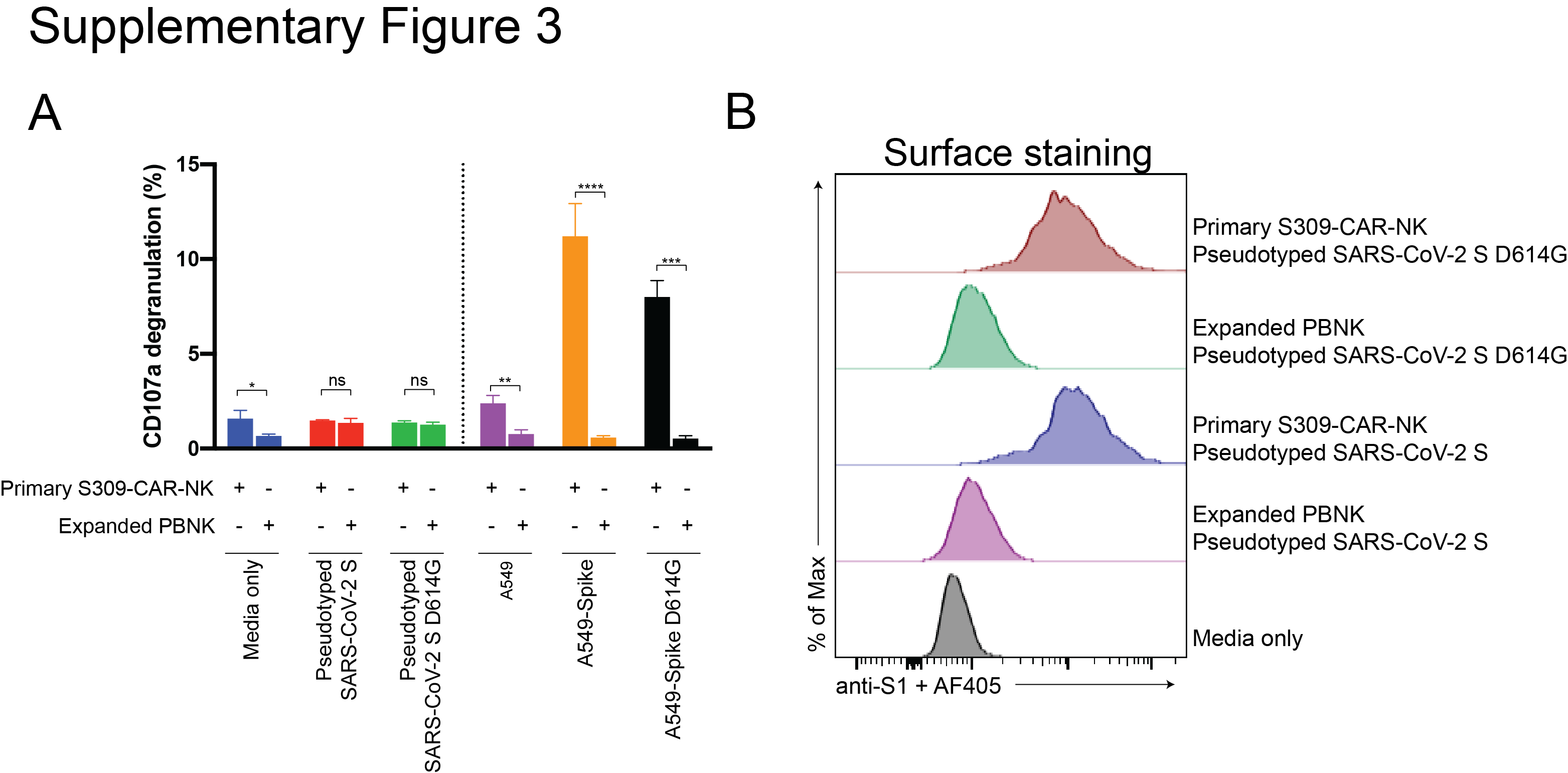

Supplement: Supplementary Figure 3 — S309-CAR-NKprimary cells is not activated upon pseudotyped SARS-CoV-2 viral particle binding. (A) Degranulation CD107a of S309-CAR-NKprimary cells upon pseudotyped SARS-CoV-2 S viral particle binding. Briefly, either expanded PBNK or S309-CAR-NKprimary cells were cocultured with media only, pseudotyped SARS-CoV-2 S, or pseudotyped SARS-CoV-2 S D614G for 2 hours at 37°C, where A549 or A549-Spike or A549-Spike D614G cells were used as positive controls, respectively. Cells were then collected and stained for anti-CD56, anti-CD3, anti-hIgG F(ab’)2, anti-CD107a, and anti-Spike subunit 1 (anti-S1) followed by flow cytometry. (B) Confirmation of pseudotyped SARS-CoV-2 viral particle binding to S309-CAR-NKprimary cells. Representative data are shown. Error bars represent means ± SD. The experiment was performed two times. [file Image_3.tiff]

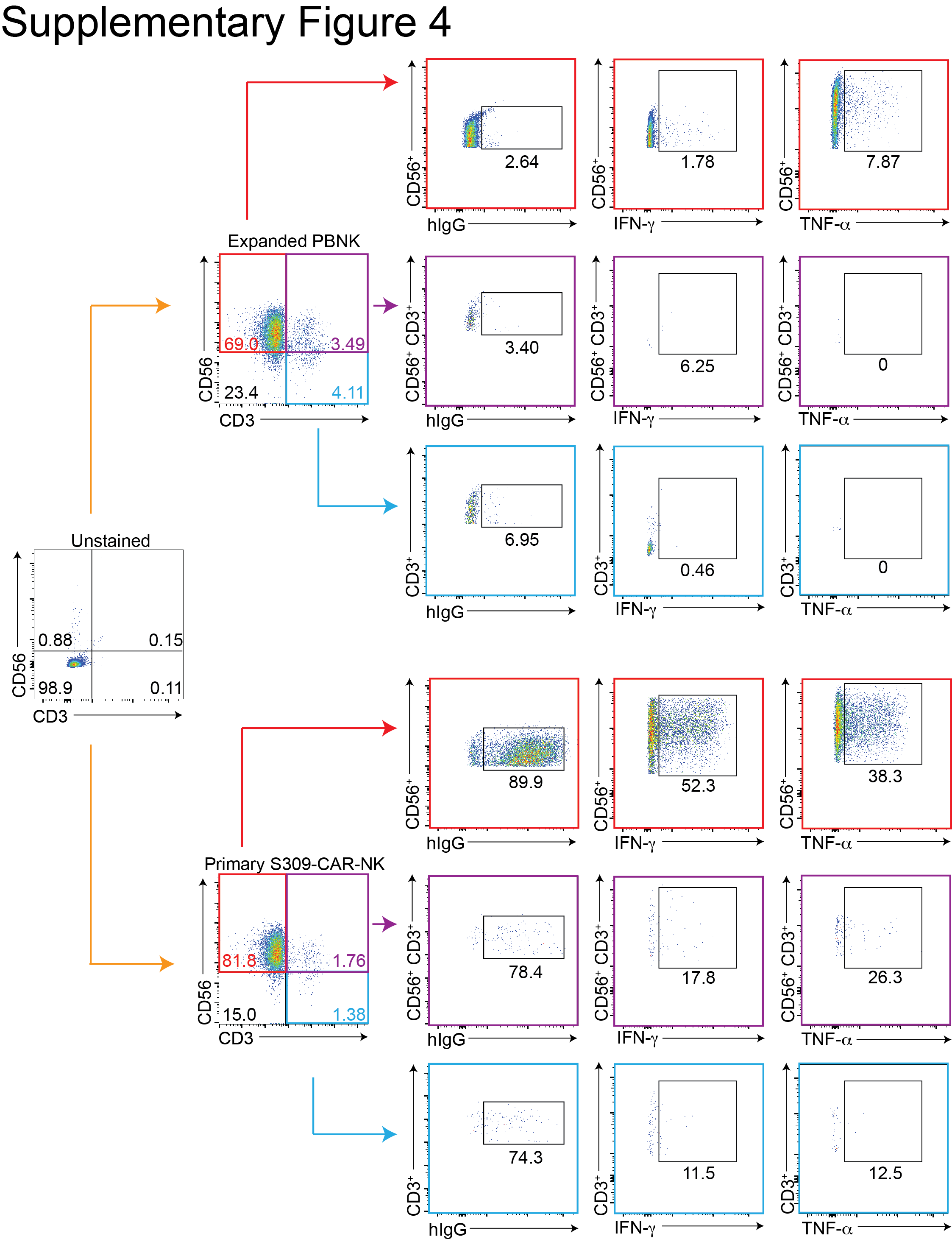

Supplement: Supplementary Figure 4 — Representative dot plots showing the INF-γ or TNF-α release of CD56+ NK cells or CD56+ CD3+ cells or CD3+ T cells. Expanded PBNK or primary S309-CAR-NKprimary cells were cocultured with A549 Spike target cells for 2 hours at 37°C in the presence of GolgiStop. Cells were subsequently stained for anti-CD56, anti-CD3, and anti-hIgG (H+L)(Fab’)2 followed by cell permeabilization and stained for anti-IFN-γ or anti-TNF-α. Flow cytometry was performed to determine the intracellular cytokine production. [file Image_4.tif]

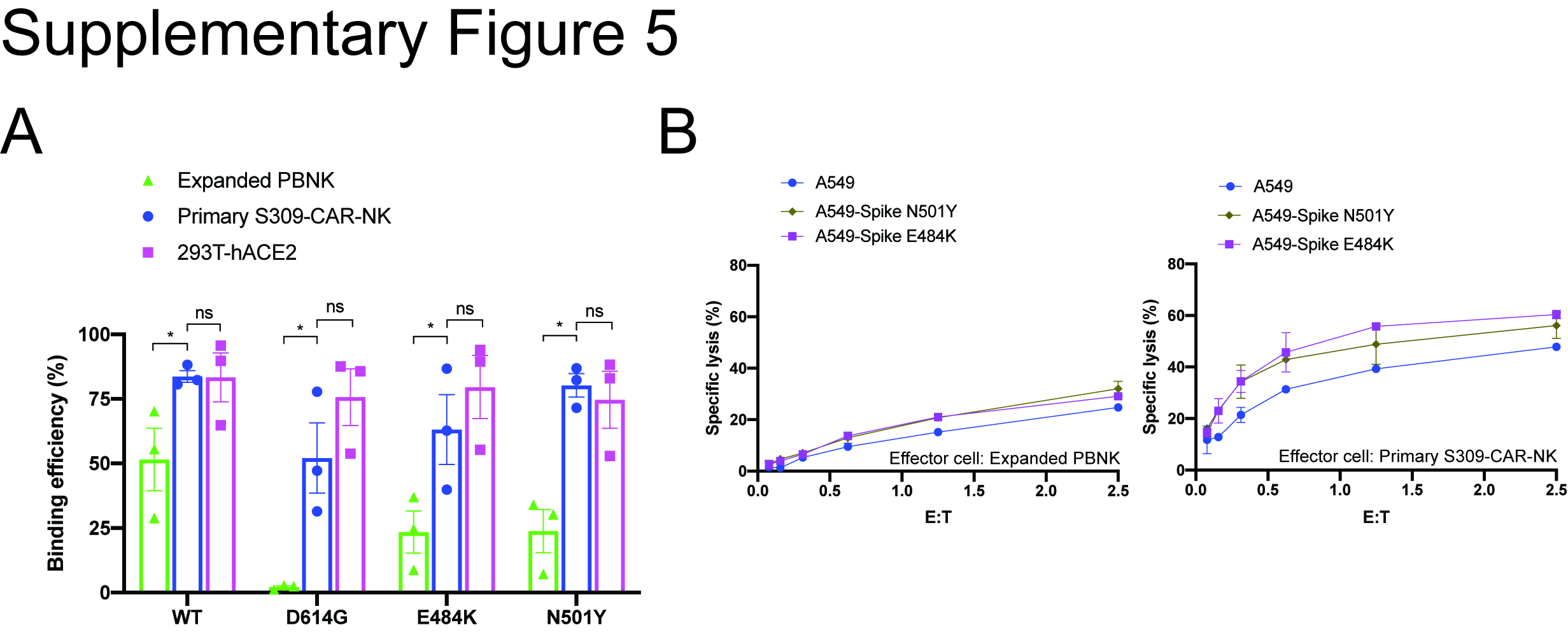

Supplement: Supplementary Figure 5 — S309-CAR-NKprimary cells are activated by other SARS-CoV-2 variants. (A) S309-CAR-NKprimary cells bind to pseudotyped SARS-CoV-2 viral particles bearing N501Y or E484K. Briefly, the pcDNA3.1-SARS-CoV-2 S plasmid was mutagenized using the primers listed in Table S1 . The pseudotyped SARS-CoV-2 viral particles were generated using the described method. S309-CAR-NKprimary or expanded PBNK or 293T-hACE2 cells were incubated with the pseudotyped SARS-CoV-2 S viral particles containing D614G, or E484K, or N501Y, and wildtype (WT) was used as a positive control, for 2 hours at 37°C. Cells were subsequently stained extracellularly for Spike with rabbit anti-RBD followed by secondary anti-rabbit prior to flow cytometry. S309-CAR-NKprimary and expanded PBNK cells were expanded from one donor. Data were pooled from three independent experiments. (B) S309-CAR-NKprimary cells directly kill A549 cells expressing Spike bearing E484K or N501Y mutation. Expanded PBNK or S309-CAR-NKprimary cells were cocultured with A549, A549-Spike E484K, or A549-Spike N501Y for 4 hours at 37°C. Samples were performed in triplicates with means ± SD. Non-parametric test was employed for panel (A). ns p > 0.05 and * p < 0.05. [file Image_5.tif]

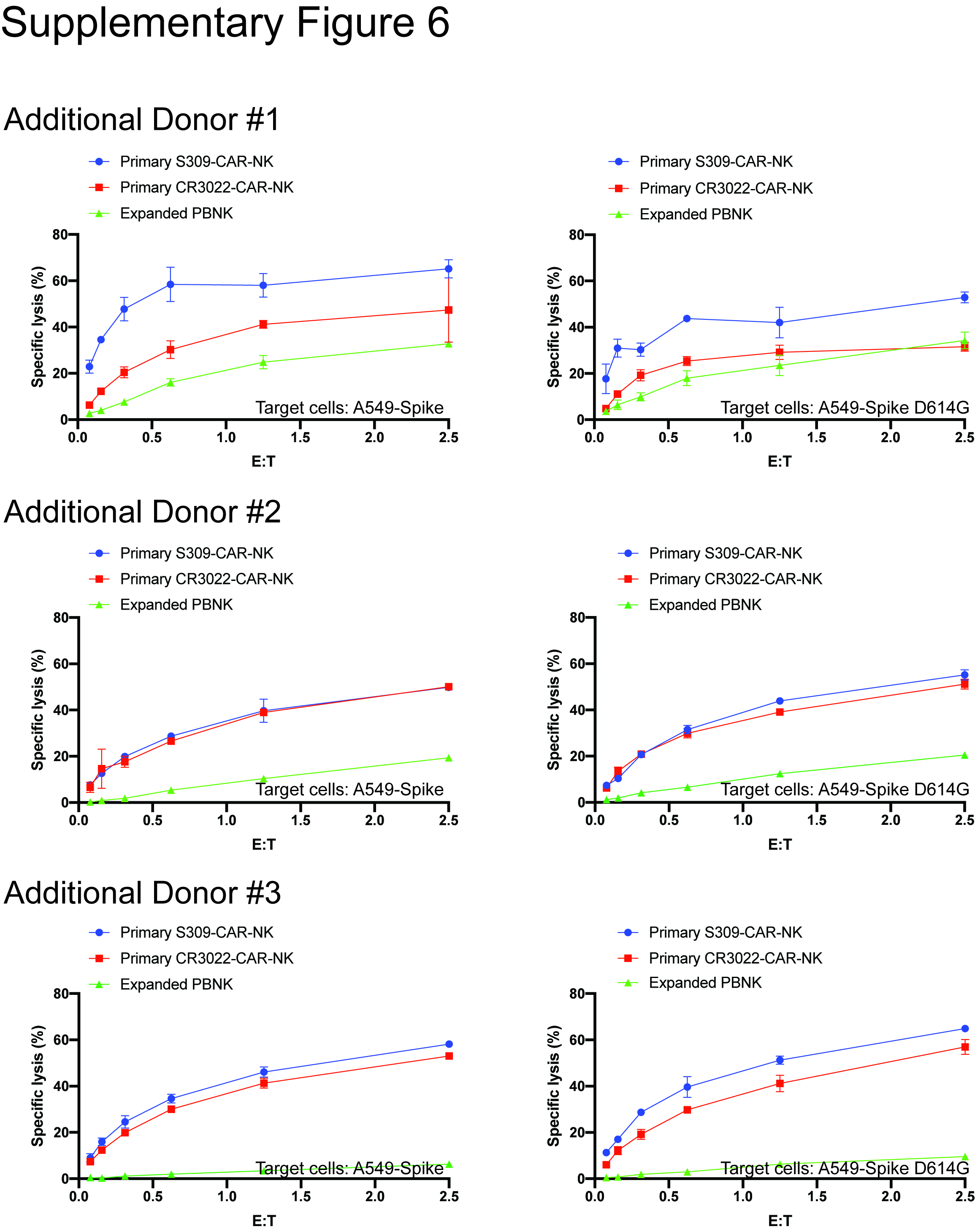

Supplement: Supplementary Figure 6 — Chromium release assay of expanded NK cells and CAR-NK cells against A549-Spike or A549-Spike D614G. Peripheral blood mononuclear cells were isolated from the buffy coats received from New York Blood Center as described in the methods section. Primary NK cells were expanded and transduced using the retrovirus packaging system to generate S309-CAR-NKprimary or CR3022-CAR-NKprimary cells from three different healthy donors. Chromium release assay was performed by coculturing effector cells with A549-Spike or A549-Spike D614G target cells at different effector: target (E/T) ratios for 4 hours at 37°C. Representative data from one experiment per donor are shown. Error bars represent means ± SD. [file Image_6.tif]
