# Supplementary material for: CAR-NK Cells Effectively Target SARS-CoV-2-Spike-Expressing Cell Lines In Vitro
Source: Front Immunol. 2021 Jul 23;12:652223. doi: 10.3389/fimmu.2021.652223 (PMC8343231; doi:10.3389/fimmu.2021.652223)
Supplement: Supplementary file 7 [file Table_1.docx]

| **Antibodies** | **Clone** | **Catalog #** | **Manufacturer** |
| --- | --- | --- | --- |
| PE anti-human CD3 | OKT3 | 317307 | BioLegend |
| FITC anti-human CD56 | HCD56 | 318303 | BioLegend |
| PE/Cy7 anti-human CD56 | HCD56 | 318317 | BioLegend |
| PE anti-human CD69 | FN50 | 310906 | BioLegend |
| APC/Fire 750 anti-human CD226 | 11A8 | 338319 | BioLegend |
| APC/Fire 750 anti-human KLRG1 | SA231A2 | 367717 | BioLegend |
| BV421 anti-human CD335 | 9E2 | 331913 | BioLegend |
| PE/Cy7 anti-human CD244 | C1.7 | 329519 | BioLegend |
| PE anti-human CD152 | BNI3 | 369603 | BioLegend |
| APC anti-human CD366 | F38-2E2 | 345011 | BioLegend |
| PerCP/Cy5.5 anti-human TIGIT | A15153G | 372717 | BioLegend |
| FITC anti-human CD223 | 11C3C65 | 369307 | BioLegend |
| APC anti-human CD94 | DX22 | 305508 | BioLegend |
| AF-700 anti-human IFN-γ | B27 | 506515 | BioLegend |
| PE anti-human TNF-α | Mab11 | 502908 | BioLegend |
| APC anti-human CD16 | 3G8 | 561248 | BD Biosciences |
| BV711 anti-human CD314 | 1D11 | 563688 | BD Biosciences |
| FITC anti-human CD107a | H4A3 | 555800 | BD Biosciences |
| PE anti-human NKG2C/CD159c | 134591 | FAB138P | R&D Systems |
| APC anti-human NKG2A | 131411 | FAB1059A | R&D Systems |
| AF647 Goat anti-human IgG(H+L) F(ab’)_2_ fragment |  | 109-136-088 | Jackson ImmunoResearch |
| Goat anti-SARS-CoV-2 Coronavirus Spike protein (subunit 1) |  | 40150-R007 | SinoBiological |
| Goat anti-SARS-CoV-2 Spike RBD |  | 40592-T62 | SinoBiological |
| Mouse anti-His | H-3 | sc-8036 | Santa Cruz Biotechnology |
| IgG (H+L) Goat anti-rabbit, AF488, Superclonal |  | PIA27034 | Fisher Scientific |
| Goat anti-Mouse IgG (H+L), Superclonal Recombinant Secondary Antibody, AF488 |  | A28175 | Fisher Scientific |

**Supplementary Table S1:**
