# Supplementary material for: CAR-NK Cells Effectively Target SARS-CoV-2-Spike-Expressing Cell Lines In Vitro
Source: Front Immunol. 2021 Jul 23;12:652223. doi: 10.3389/fimmu.2021.652223 (PMC8343231; doi:10.3389/fimmu.2021.652223)
Supplement: Supplementary file 8 [file Table_2.docx]

| **Construct name** | **Primer name** | **Primer sequence** |
| --- | --- | --- |
| **pSFG-SARS-CoV-2 S** | SFG-SARS-CoV-2-S.For | 5’-TCTAG AGATT ACAAG GATGA CGACG ATAAG TAACT CGAGA TCGAT CCGGA TTAGT CCAAT- 3’ |
|  | SFG-SARS-CoV-2-S.Rev | 5’-GTCGA CGCAC TGGAC ACCTT TTAAA ATAG- 3’ |
| **pSFG-SARS-CoV-2 S D614G** | SFG-SARS-CoV-2-S D614G.For | 5’-GTGCT GTACC AGGGC GTGAA TTGCA C- 3’ |
|  | SFG-SARS-CoV-2-S D614G.Rev | 5’-GGTTA TGGTC GTTGG TCCAC CGG- 3’ |
| **pSFG-SARS-CoV-2 S E484K** | SFG-SARS-CoV-2-S E484K.For | 5’ -AAGGG CTTCA ACTGC TACTT CCCCC TG- 3’ |
|  | SFG-SARS-CoV-2-S E484K.Rev | 5’ -CACTC CATTG CATGG GGTGC TTCCA G- 3’ |
| **pSFG-SARS-CoV-2 S N501Y** | SFG-SARS-CoV-2-S N501Y.For | 5’ -TACGG AGTGG GATAC CAGCC ATACA GG- 3’ |
|  | SFG-SARS-CoV-2-S N501Y.Rev | 5’ -GGTTG GCTGG AAGCC GTAGC TCTG- 3’ |
